# Supplementary material for: Microfluidics-Assisted Size Tuning and Biological Evaluation of PLGA Particles
Source: Pharmaceutics. 2019 Nov 8;11(11):590. doi: 10.3390/pharmaceutics11110590 (PMC6921086; doi:10.3390/pharmaceutics11110590)
Supplement: Supplementary file 1 [file pharmaceutics-11-00590-s001.pdf]

Microfluidics-Assisted Size Tuning and Biological Evaluation of PLGA Particles

Maria Camilla Operti [1], Yusuf Dölen [1,2], Jibbe Keulen [1], Eric A. W. van Dinther [1,2], Carl G. Figdor [1,2] and Oya Tagit [1,2,*]

[1] Department of Tumor Immunology, Radboud Institute for Molecular Life Sciences, Radboud University Medical Center, 6500 HB Nijmegen, The Netherlands; MariaCamilla.Operti@radboudumc.nl (M.C.O.); Yusuf.Dolen@radboudumc.nl (Y.D.); jibbekeulen@gmail.com (J.K.); Eric.vanDinther@radboudumc.nl (E.A.W.v.D.); Carl.Figdor@radboudumc.nl (C.G.F.)

[2] Oncode Institute, 3553 Utrecht, the Netherlands

* Correspondence: oya.tagit@radboudumc.nl

Supplementary Information

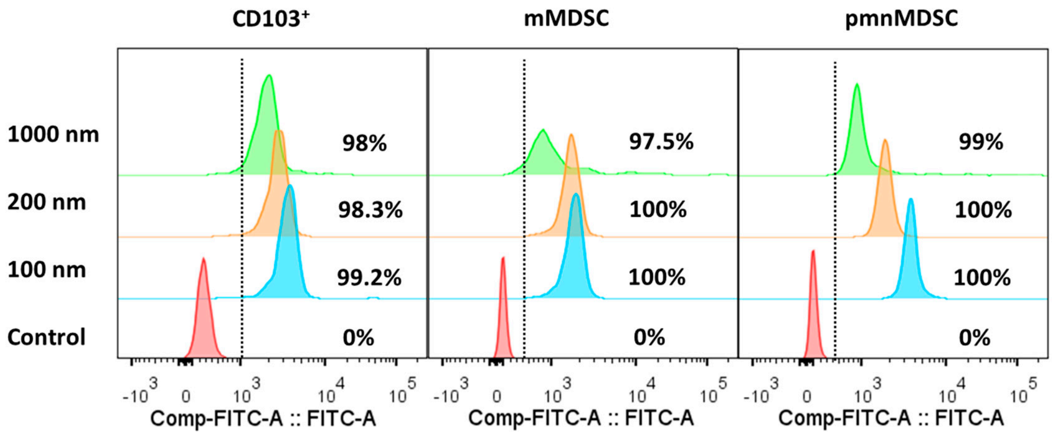

Figure S1. Flow cytometry histograms of the uptake experiments. Percentage of cells that contain fluorescently labeled particles are shown on the plots. For all cases, more than 97% of the cells contain the fluorescent particles. A clear correlation between the particle size and cellular fluorescence intensity is observed. Since the number of particles incubated with cells was adjusted for equal fluorescence intensities, the observed variation of cellular fluorescence levels can be directly correlated with particle uptake efficiency, which is less for micron-size particles.

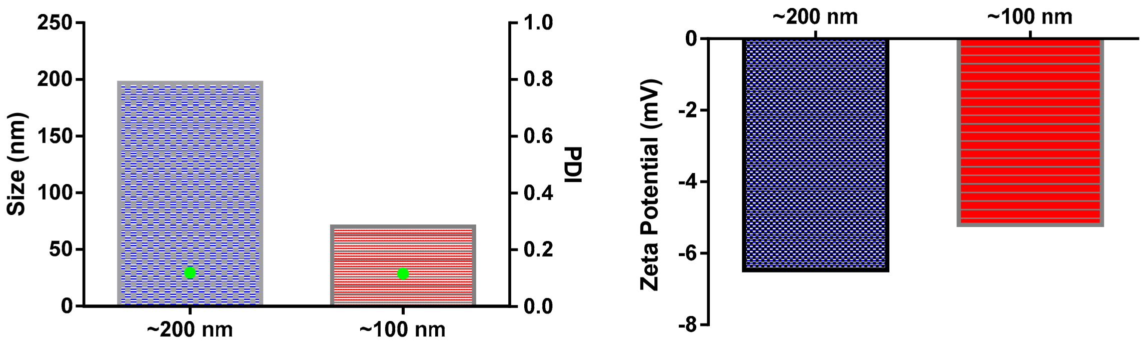

Figure S2. Size distribution (left) and ζ potential values (right) of PLGA particles encapsulating a NIR-emitting fluorescent dye.

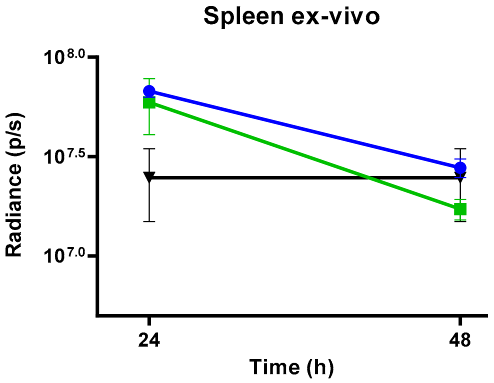

Figure S3. Variations of spleen fluorescence intensities of ~100 nm (blue) and ~200 nm (green) PLGA particles obtained on an isolated spleen at 24 h and 48 h after particle administration. Data obtained for untreated mice (negative control) are shown in black.
